# Glycosaminoglycan-Protein Interactions and Their Roles in Human Disease

**DOI:** 10.3389/fmolb.2021.639666

**Published:** 2021-03-09

**Authors:** Deling Shi, Anran Sheng, Lianli Chi

**Affiliations:** National Glycoengineering Research Center, Shandong University, Qingdao, China

**Keywords:** glycosaminoglycan, protein, human disease, interaction, molecular recognition

## Abstract

Glycosaminoglycans (GAGs) are a family of linear and negatively charged polysaccharides that exist ubiquitously on the human cell surface as well as in the extracellular matrix. GAGs interact with a wide range of proteins, including proteases, growth factors, cytokines, chemokines and adhesion molecules, enabling them to mediate many physiological processes, such as protein function, cellular adhesion and signaling. GAG-protein interactions participate in and intervene in a variety of human diseases, including cardiovascular disease, infectious disease, neurodegenerative diseases and tumors. The breakthrough in analytical tools and approaches during the last two decades has facilitated a greater understanding of the importance of GAG-protein interactions and their roles in human diseases. This review focuses on aspects of the molecular basis and mechanisms of GAG-protein interactions involved in human disease. The most recent advances in analytical tools, especially mass spectrometry-based GAG sequencing and binding motif characterization methods, are introduced. An update of selected families of GAG binding proteins is presented. Perspectives on development of novel therapeutics targeting specific GAG-protein interactions are also covered in this review.

## Introduction

Recently, COVID-19 disease, caused by severe acute respiratory syndrome-related coronavirus 2 (SARS-CoV-2), has led to medical and economic disruptions worldwide. Reports have shown that heparan sulfate (HS) is an indispensable cofactor for SARS-CoV-2 infection by interacting with both SARS-CoV-2 spike glycoprotein and angiotensin-converting enzyme 2 (ACE2) in the receptor-binding domain (RBD) (Clausen et al., 2020; Kim et al., 2020). Evidence has shown that heparin and its derivatives may contribute to the fight against SARS-CoV-2 infection and side effects (Liu et al., 2020; Tandon et al., 2020) by targeting the interaction between HS and related proteins. These studies have emphasized the importance of the interactions between glycosaminoglycans (GAGs) and proteins in disease and their roles as novel therapeutic targets, these interactions have been studied for decades but still lag behind the study of protein-protein and protein-nucleic acid interactions due to the structural complexity of GAGs and limitations of analytical tools.

GAGs are a family of linear and negatively charged polysaccharides that are commonly expressed in the interior and surrounding environment of most cell types, with a molecular weight of approximately 10–100 kDa (Kowitsch et al., 2018). Among the naturally occurring polysaccharides, the structure of GAGs is extremely complex due to alterations in residue types, glycosidic bond types, sulfation levels, sulfation positions and chain lengths. According to the type of hexosamine, hexose or hexuronic acid in the disaccharide repeating units and the glycosidic linkage between these units, GAGs are divided into five main types: nonsulfated GAGs, such as hyaluronic acid (HA) (Dymarska et al., 2016), and sulfated GAGs, including heparin and HS (Shriver et al., 2012), chondroitin sulfate (CS) (Purushothaman et al., 2012), dermatan sulfate (DS) (Yamada and Sugahara, 2008), and keratan sulfate (KS) (Pomin, 2015). Heparin (∼2.3 sulfate groups per disaccharide) and HS (∼0.8 sulfate groups per disaccharide) consist of basic disaccharide repeats (GlcA/IdoA*β*1-4GlcNAc*α*1-4) _n_, while the 3- and 6-positions of the glucosamine residue or the carboxyl group of uronic acid may be substituted or not substituted with sulfate groups. Heparin and HS have received the most attention and have been studied extensively due to their high sulfation and diverse biological activities, which are also our first concerns herein. Except for HA, all mammalian GAGs are linked to a core protein to form proteoglycans (PGs). The structure of the protein cores, the composition of the glycosaminoglycan chains, and the distribution of the proteoglycan all affect the biological activity of proteoglycans (Lindahl et al., 2015).

**FIGURE 1:**
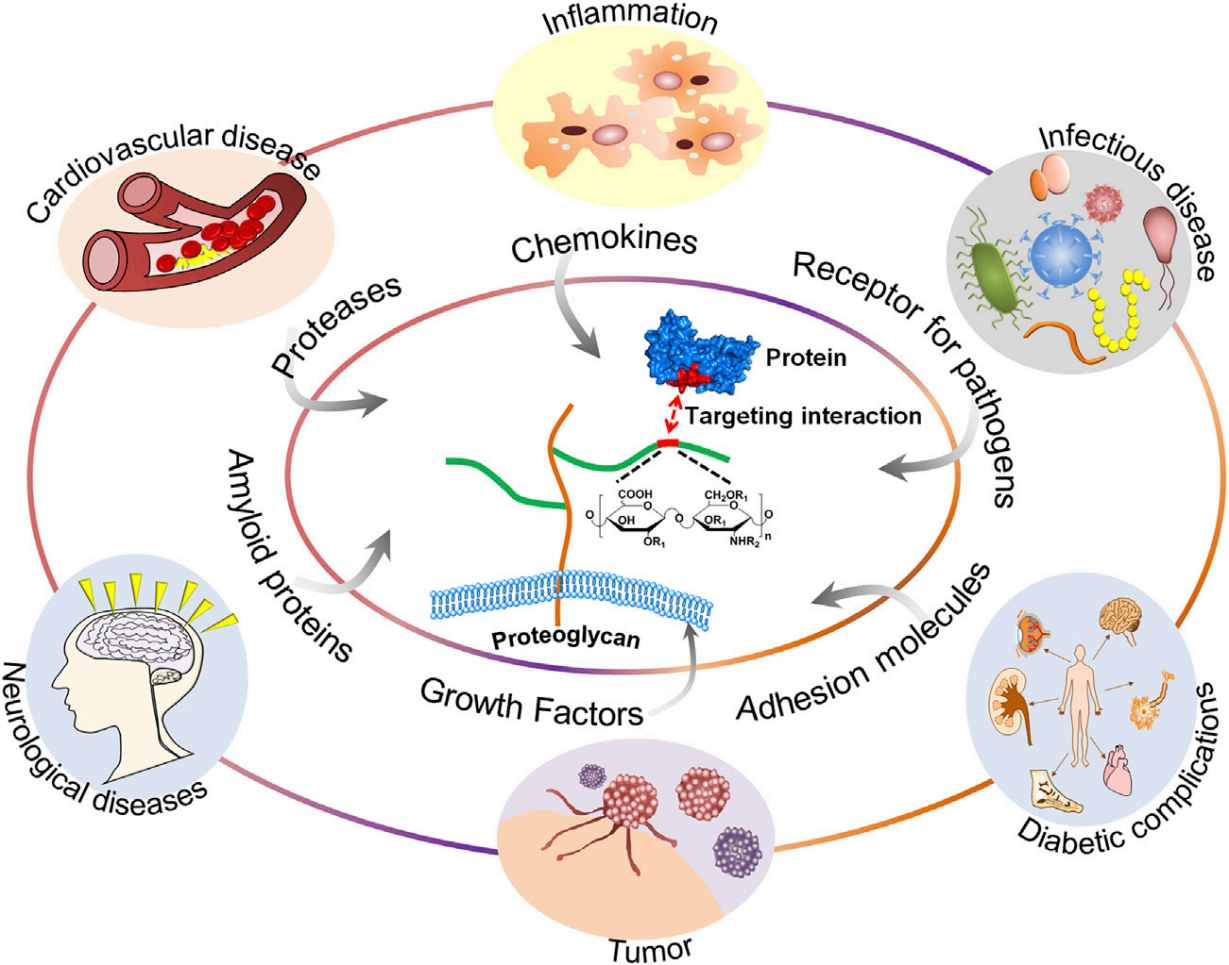
A schematic representation of the structure of GAGs and their interactions with proteins and functions relevant to specific diseases.

GAGs are of vital importance in the field of glycobiology, especially their multiple roles as signal molecules that regulate protein activity and act as structural components and effectors of cellular activity. GAGs have been demonstrated to modulate numerous biological processes, ranging from embryonic development, regulation of enzymatic activities, extracellular matrix assembly, and ligand binding to receptors to the regulation of cell signaling, through the regulation of distinct proteins, such as growth factors, chemokines, and adhesion molecules (Vallet et al., 2021). These processes are particularly important when related to diseases, including cardiovascular disease (Wight, 2018), cancer (Ma et al., 2020), infectious diseases (Kamhi et al., 2013), neurodegenerative diseases (Huynh et al., 2019), inflammatory responses (Morla, 2019), and wound healing (Salbach et al., 2012). A schematic representation of the structure of GAGs and their interactions with proteins and functions relevant to specific diseases is shown in Figure 1.

**FIGURE 2:**
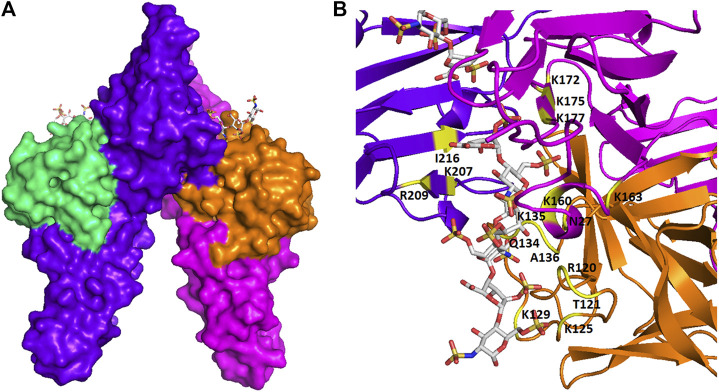
The spatial structure of an FGF-FGFR-heparin complex. **(A)** The surface view. **(B)** The view of ribbon structure. The heparin fragments (ΔUA-GlcNS6S-IdoA2S-GlcNS6S-IdoA2S- GlcNS6S) that make contacts to two FGF2s (shown in green and orange) and two FGFR1s (shown in purple and red) are represented as balls and sticks. The amino acid residues that participate in the interaction are indicated. The figure was prepared by using PDB code 1FQ9, which was originally reported in the reference Schlessinger et al. (2000).

The binding between GAGs and proteins are prominently ionic. Non-ionic forces, including hydrogen bonding and hydrophobic interaction, sometimes also play a major role in forming the GAG-protein complexes (Capila and Linhardt, 2002). It has been controversial that the binding between GAGs and proteins are non-specific until recently, as more and more studies have revealed the relatively high selectivity of GAG sequence to specifically bind to certain proteins. The binding posture and specificity were demonstrated in Figure 2, using a fibroblast growth factor (FGF)- FGF receptor (FGFR)-heparin complex as an example (Schlessinger et al., 2000). Furthermore, FGF1, and FGF2 signaling through FGFR 1c showed clearly different specificity when screening against a library of chemoenzymatically synthesized HS with defined structures (Schultz et al., 2017). Additional examples on specificity of GAG-protein interactions include a 2-*O*-sulfate-GlcA containing HS hexasaccharide selectively activating heparin cofactor II (Sankarayanarayanan et al., 2017), a 3-*O*-sulfated HS being preferentially recognized by SARS-CoV-2 spike glycoprotein (Tiwari et al., 2020), and a 3-*O*-sulfated HS octasaccharide specifically binding to herpes simplex virus type 1 glycoprotein D (Huang et al., 2017). Besides, high-throughput study using HS microarray revealed that HS-binding proteins, including FGF2 and several chemokines, require clearly different ligands on HS (Zong et al., 2017). A review focused on the topic of selectivity of GAG-protein interactions has been recently written by Kjellén and Lindahl (Kjellen and Lindahl, 2018). The selectivity of these interactions is fundamental for designing HS mimetics as promising therapeutics.

There is increasing interest in exploring the essentials of GAG-protein interactions and their roles in human diseases. In particular, novel therapeutics targeting specific GAG-protein interactions have important application value, such as the treatment of coronary pneumonia. As the interaction between GAGs and proteins involves a wide range of physiological processes, the influence of their interaction on specific diseases and their potential therapeutic effects have attracted much attention in an effort to find new methods for treatment or prevention of disease. To synthesize structural analogs, remove or modify structures, or block the interaction with reagents, it is necessary to obtain defined mechanisms and binding sequences. The recent breakthroughs in analytical tools and approaches, especially mass spectrometry (MS)-based GAG sequencing and binding motif characterization methods, have facilitated a greater understanding of the structural basis and mechanisms of GAG-protein interactions, creating an opportunity to utilize the structural diversity of GAGs to discover novel therapeutics. Further understanding of the interaction process and mechanism between GAGs and proteins will contribute to the proper understanding of the occurrence and development of a great number of diseases and the development of new therapeutic approaches.

This review focuses on the interaction between GAGs and proteins and their effect in human disease. In addition, the molecular basis and mechanisms of GAG-protein interactions are introduced. The latest progress in GAG-binding proteins and analytical tools is also discussed. Moreover, perspectives on development of novel therapeutics targeting specific GAG-protein interactions are presented.

## Human Diseases Related to GAG-Protein Interactions

### Cardiovascular Disease

The first specific GAG-protein interaction described was heparin and antithrombin, which has important physiological significance and was used in the production of pharmaceutical heparin products as anticoagulants for treatment of thrombosis, embolism and thrombophlebitis. Heparin and low molecular weight heparin (LMWH) inhibit coagulation factors Xa and IIA by combining with antithrombin III to prevent thrombosis. Since then, the function of GAGs and their interaction with proteins in the vascular system have been studied. Although heparin is successfully used to prevent thrombosis in hospitalized patients, it was reported to present a risk of bleeding at prophylactic doses (Sunseri et al., 2018). This prothrombotic adverse reaction, named heparin-induced thrombocytopenia (HIT), is mediated by immunity and is also caused by an interaction between heparin and protein. Heparin products form multimolecular complexes with antigenic platelet factor 4 (PF4), resulting in the formation of IgG platelet-activating antibodies which are against the heparin/PF4 complex, which triggers an immune response and induces platelet activation and aggregation (Ho and Siordia, 2016). This process leads to platelet reduction and thrombin generation, ultimately resulting in thrombocytopenia. At the same time, the process may also be accompanied by the formation of venous or arterial thrombosis, which then develops into deep venous thrombosis and pulmonary embolism (Warkentin, 2018).

Early studies have shown that GAGs accumulate in disease-prone areas of the vascular system, such as at branch points, and are often consistent with lipid deposition. Subsequent studies have shown that GAGs are covalently linked to specific core proteins and interact with different ligands within the interstitial space to help regulate vascular structure and function. PGs also interact with a variety of receptors on the surface of vascular cells, partially regulating the phenotype of vascular cells (Wight, 2018). For example, DSPG can promote the formation of atherosclerosis (Edwards et al., 2004), while CSPG may participate in the process of early atherosclerosis intimal thickening (Wight and Merrilees, 2004). HSPG is negatively regulated by atherogenic molecules; thus, the lipoprotein regulation of endothelin may play a key role in the formation of atherosclerosis (Pillarisetti, 2000). Recently, the relationship between cardiovascular disease and heparin-binding protein (HBP) was confirmed by using bioinformatics methods (Cai et al., 2020), which showed that HBPs may act as a novel biomarker linking cardiovascular diseases, such as atherosclerosis, myocarditis, myocardial ischemia, and myocardial infarction (MI). Specific HBPs or signaling pathways can be developed as new therapies for cardiovascular disease.

### Tumors

In the last few decades, PGs have been found to be involved in the functions and mechanisms of cancer cells and play a key role in cancer cell adhesion, migration, invasion, and metastasis. HS proteoglycans (HSPGs) are proteins that are covalently linked with HS. The main HSPGs can be classified into two main categories: cell surface HSPGs (syndecans and glypicans) and basement membrane HSPGs (perlecan, agrin and collagen type XVIII). HSPGs are downregulated or upregulated in different tumors (De Pasquale and Pavone, 2020). GPC1, a cell surface HSPG, was found to be overexpressed in breast cancer (Matsuda et al., 2001), glioma (Saito et al., 2017), and pancreatic cancer (Kleeff et al., 1998) but downregulated in colorectal cancer (Knelson et al., 2014). HS can bind growth factors to regulate angiogenesis, including fibroblast growth factors (FGFs), vascular endothelial growth factors (VEGFs) and platelet-derived growth factors (PDGFs). Perlecan on the tumor cell surface can interact with ligand and adaptor proteins to enhance FGF signaling and tumor angiogenesis (Whitelock and Iozzo, 2005). If the C-terminus of perlecan is lacking, VEGF synthesis would be reduced to suppress tumor angiogenesis (Sharma et al., 1998). The other GAGs also have important functions in tumors. CS-E is not expressed in normal ovaries or cystadenomas but is highly expressed in extracellular matrices (ECMs) of ovarian adenocarcinomas to mediate VEGF binding (Ten Dam et al., 2007). It has been reported that the tumor microenvironment can induce HA production (Tammi et al., 2011). HA is highly expressed in breast cancer (Auvinen et al., 2000), lung cancer (Pirinen et al., 2001) and ovarian cancer (Anttila et al., 2000), while HA expression is low in squamous cell carcinoma and melanoma (Karjalainen et al., 2000; Kosunen et al., 2004). From these studies, the abnormal PG expression levels or structural changes in PGs during tumorigenesis and progression indicate their importance as potential biomarkers of cancer occurrence and progression and as therapeutic targets.

### Infectious Disease

Given their ubiquity and abundant biological functions, GAGs are the main target of pathogens in the infection process and play an important role in the initial attachment of pathogens to host cells. Studies have shown that GAGs interact with microbial pathogens on the cell surface and ECMs to modulate microbial pathogenesis and host defense. Many pathogenic microorganisms, such as viruses (e.g., human papilloma virus (HPV) (Kines et al., 2009), hepatitis C virus (HCV) (Barth et al., 2003), dengue virus (Dalrymple and Mackow, 2011), bacteria (e.g., *Listeria monocytogenes* (Banerjee et al., 2004) and protozoa (e.g., malaria sporozoites (Clausen et al., 2012) can express proteins that bind to HS, DS, and CS on cell surfaces, thereby facilitating the host cell infection process.

The latest evidence shows that HS, as a cofactor of SARS-CoV-2 infection, transforms the spinous process structure into an open conformation through interaction of the spike glycoprotein in the RBD of SARS-CoV-2 to promote the binding of adjacent ACE2 (Clausen et al., 2020). Previous experiments have shown that HSPGs are essential cell-surface molecules involved in SARS-CoV cell entry by providing binding sites for SARS-CoV invasion at the early stage (Lang et al., 2011). Coronavirus NL63 entry into host cells relies on HS interactions that increase virus density at the cell surface. The entry of coronavirus NL63 into host cells is achieved by using GAGs as adhesion molecules to increase the virus density on the cell surface, which is an example of pathogens using GAGs to survive (Milewska et al., 2014). Other microbial pathogens, such as Middle East respiratory syndrome coronavirus (MERS-CoV) and the Gram-negative bacterium *Pseudomonas aeruginosa* (Park et al., 2001), which can cause respiratory infections, have also been reported to interact with GAGs.

When the skin barrier is damaged, the GAGs at the wound site will change and can bind to pathogens, such as Merkel cell polyoma virus (MCV) (Schowalter et al., 2011), *S. aureus* (Liang et al., 1992), *Candida* (Green et al., 2013) and *Leishmania* (Fatoux-Ardore et al., 2014). Merkel cell polyoma virus (MCV) infection is an example. MCV is a circular double-stranded DNA virus and the causative agent of Merkel cell carcinoma, which is a rare but fatal skin cancer. When MCV first attaches to cells, it mainly binds to HS on the cell surface and, to a lesser extent, binds to CS. After treatment of cells with heparanase and chondroitinase sulfate, MCV infection is significantly affected. In addition, other diseases are related to the interaction of GAGs and pathogenic microorganisms, including enterocolitis (Boyd et al., 1998), diarrhea (Viboud and Bliska, 2005), keratitis (Hayashida et al., 2011), and AIDS (Hayashida et al., 2015).

### Diabetic Complications

Diabetes encompasses a group of lifelong metabolic diseases characterized by chronic hyperglycemia due to multiple causes. According to World Health Organization statistics, diabetes is the disease with the most complications, including diabetic cardiopathy, diabetic ocular surface, and diabetic foot. One of the most important complications for diabetic patients is diabetic nephropathy. Diabetic nephropathy is a major microvascular complication in long-term diabetic patients. The prolonged hyperglycemia caused by diabetes can lead to glycosylation and non-enzymatic cross-linking between proteins and glucose or its derivatives (Qiu et al., 2020). A series of further complex molecular rearrangements produces irreversible advanced glycation end products (AGEs). AGEs initiate and accelerate the development of renal disease by activating the receptor for advanced glycation end products (RAGE). Through surface plasmon resonance (SPR) analysis, it was found that the affinity of RAGE for low molecular weight heparins (LMWHs) was approximately 6 times higher than that for AGEs. The antagonistic effect of LMWHs on RAGE helps to improve diabetic nephropathy (Myint et al., 2006). A permeability change in the capillary wall of the glomerulus is an early manifestation of diabetic nephropathy, which clinically manifests as abnormal proteinuria. The basement membrane of the glomerulus contains highly negatively charged GAGs represented by HS, which can prevent passage of charged macromolecules. Neutralization of anions in the capillary wall of the glomerulus is related to the loss of charge-dependent glomerular permeability selectivity. The decrease in HS is due to the increase in heparanase-1 gene expression in glomerular epithelial cells induced by glucose in patients with diabetic nephropathy. Heparin or LMWHs can be used as heparinase inhibitors to effectively reverse the abnormal permeation selectivity of the glomerulus and improve diabetic nephropathy (Lewis and Xu, 2008).

### Mucopolysaccharidoses

Mucopolysaccharidoses (MPS) are a group of diseases caused by abnormal accumulation of GAGs. The patients are of genetic defects and produce no or deficient lysosomal enzymes to degrade metabolic GAGs. Based on the deficient enzyme and symptom, MPS are divided into seven different types and more subtypes. Unfortunately, there is no medical treatment can cure these diseases. Most studies are focused on the early diagnostics of MPS. Currently, enzyme replacement therapy and hematopoietic stem cell transplantation are primarily used in clinic to control the progress of MPS and improve the conditions of patients (Zhou et al., 2020).

In MPS patients, GAGs are accumulated in cells, blood and tissues, which consequents to pathological symptoms over time. However, the exact mechanism of biological interactions with accumulated GAGs and proteins remains unclear. Most recent research in this filed suggested that abnormally accumulated HS in MPS patients tightly bound to cathepsin V and inhibited its elastolytic activity. HS antagonist was able to restore the activity of cathepsin V (Chazeirat et al., 2021). The new findings encourage exploring novel approaches for treating MPS and associated disorders based on the molecular interaction between GAGs and proteins.

### Other Diseases

GAGs also play a crucial role in inflammation, neurological diseases (e.g., Parkinson’s disease, Alzheimer's disease (AD), and mad cow disease) and other diseases. The important role of GAGs in the inflammatory response has been reported in previous studies. As the structural heterogeneity of HS is usually concentrated in the high-sulfate region, it can participate in almost every stage of leukocyte passage through the vascular wall and can interact with a variety of proteins, such as L-selectin, CXC-chemokine ligand 8 (CXCL8), and histidine-rich glycoprotein (HRG) (Parish, 2006). The interaction of HA with CD44 and tumor necrosis factor-stimulated gene-6 (TSG-6) activates a variety of inflammatory cells (Baranova et al., 2011), and HA also interacts with Toll-like receptor four to promote the release of cytokines by dendritic cells (Taylor and Gallo, 2006). LMWHs can combine with tumor necrosis factor (TNF) and the nuclear transcription factor NF-kB to prevent leukocyte extravasation (Luan et al., 2014). Moreover, some studies have shown that GAGs may be used to treat AD and other age-related dementias. GAGs can interact with basic fibroblast growth factor (FGF-2), VEGF, brain-derived neurotrophic factor (BDNF) and tau growth factors (Huynh et al., 2019). Heparin can inhibit the activity of β-site APP cleaving enzyme (BACE1) to reduce β-amyloid protein content (Cui et al., 2011). Similarly, CS extract from *Sardina pilchardus* can also inhibit BACE1 (Mycroft-West et al., 2020). In addition, GAGs are of great value in the treatment of sinusitis, asthma, chronic obstructive pulmonary disease, cystic fibrosis, and primary ciliary dyskinesia. For example, TSG-6, CD44, and Toll-like receptor 4 (TLR4) can be activated by HA, leading to calcium channel activation and immune activation (Garantziotis et al., 2016). In addition, a reduction in contractile protein content in the diaphragm and some growth factors has been reported to lead to changes in glycosaminoglycan epitopes in patients with chronic obstructive pulmonary disease (Ottenheijm et al., 2007).

As summarized herein, nearly all types of major human diseases are related to GAGs more or less. There are still great demands for therapeutics to treat these diseases. Understanding the role of GAGs in these diseases and knowing how to modulate these physiological or pathological processes using artificial GAGs might open an era of discovering new drugs based on GAGs or targeting GAGs.

## Glycosaminoglycan-Binding Proteins

### Serpins

Serpin family protein proteinase inhibitors play a critical role in regulating proteinases in diverse physiologic processes by regulating the activity of serine and cysteine proteinases through a conformational trapping mechanism, providing a finely tuned time- and location-dependent regulation of proteinase activity (Huntington, 2006). In plasma, antithrombin III (AT III) and heparin cofactor II (HC II) are major heparin-dependent protease inhibitors that maintain blood fluidity by interacting with cell surface GAGs. Antithrombin, in cooperation with heparin and HS, causes anticoagulation by preventing activation of blood clotting proteinases at the site of vascular injury. Under normal conditions, antithrombin inhibits blood clotting proteinases in a repressed reactivity state because the exposed reactive center loop (RCL) of serpin only provides the minimum specificity determinants to identify thrombin, factor Xa and factor IXa. In addition, unfavorable interactions diminish the favorable RCL and exosite interactions with proteinases. The combination of specific heparin or HS with antithrombin can induce allosteric activation, thus reducing adverse interactions and promoting template bridging of the serpin and proteinase (Olson et al., 2010). The defined protein-binding motif and molecular basis for the anticoagulant function of heparin have been reported to involve a specific pentasaccharide sequence that can bind to AT III. At least 16 saccharides of the heparin chain are required, although only the pentasaccharide is necessary (Guerrini et al., 2014). By interacting with AT III, heparin enhances AT III-mediated inhibition of thrombin and factor Xa. Inactivation of these proteases by AT III is greatly accelerated by the binding of heparin, increasing the bimolecular rate constant by a factor of 2000 (Rosenberg and Damus, 1973). Interestingly, heparin also binds to HC II but does not exhibit selectivity. Instead, the sequence of a unique DS hexasaccharide has been elucidated to interact with HC II of high affinity (Maimone and Tollefsen, 1990; Raghuraman et al., 2010). These again demonstrated the selectivity of binding between GAGs and proteins. However, Other serpins that rely on binding to GAGs to enhance their inhibition include heparin cofactor II, protein C inhibitor and protease nexin I (Munoz and Linhardt, 2004; Rein et al., 2011).

### Growth Factors

HSPGs interact with growth factors [e.g., FGFs (Huynh et al., 2019), VEGF (Gitay-Goren et al., 1992), transforming growth factor *β* (TGF-*β*) (Lee et al., 2015), and PDGF (Fager et al., 1992)] to promote their biological activities. The proteins in the FGF family may be the most extensively studied heparin-binding proteins and have a high affinity for cell surface HSPGs. FGFs participate in developmental and physiological processes through binding cell surface FGFRs as well as GAGs. These growth factors, such as acidic fibroblast growth factor (FGF-1) and FGF-2, must interact with and be activated by an active ternary complex comprising canonical receptors (FGFRs) and GAGs on endothelial surface PGs. Then, the three components FGF, FGFR, and HS interact simultaneously with signal transduction, thus triggering cell division and further processing (Fannon et al., 2000). In addition, the GAG interaction is necessary to stabilize the FGF-FGFR complex by balancing the surface charges. This interaction also limits the activity of growth factors to a certain extent. In fact, FGF binding is achieved through selected sequences (protein-binding motifs) within the HS backbone, although the minimal binding sequences are still controversial (Pomin, 2016). Heparin-binding epidermal growth factor-like growth factor (HB-EGF) is a member of the EGF family of growth factors and interacts with the EGF receptor to exert mitogenic activity in various cell types. HB-EGF is considered to play a key role in advanced brain functions in the central nervous system (Oyagi and Hara, 2012), as well as in tumor formation and other biological processes (Tsujioka et al., 2011).

### Chemokines

Chemokines are a family of small cytokines that can be classified into four groups, CXC, CC, C, and CX3C, according to their shared structural characteristics and four cysteine residues in conserved locations. Some chemokines can be induced during an immune response to promote cells of the immune system to reach the infection site, while others participate in controlling the migration of cells during normal tissue maintenance or development processes (Mantovani et al., 2006). These proteins interact with G protein-linked transmembrane receptors (called chemokine receptors) to exert their biological effects, including selective recruitment and activation of cells during inflammation, stimulation of leukocyte degranulation, and promotion of angiogenesis or angiostasis (Crijns et al., 2020). Locally produced chemokines bind to their chemokine receptors and induce leukocytes to adhere to endothelial cells, followed by extravasation of the leukocytes and subsequent migration to inflammation sites. To expose to the endothelial layer of blood vessels and form a concentration gradient, chemokines must bind to GAGs in endothelial cells and tissues (Johnson et al., 2005). In addition to PF4, which can lead to HIT, other important members of the chemokine family (e.g., stromal cell derived factor-1a (SDF-1a) and monocyte chemoattractant protein-1 (MCP-1) also bind to heparin, although with varying affinity and specificity. For example, studies have shown that HS is involved in binding and localization of SDF-1a to the cell surface. The sulfated–acetylated–sulfated domain of HS has subsequently been found to be recognized by a number of chemokines, such as IL-8, PF4 and MIP-1a (Gandhi and Mancera, 2008). Increasing evidence has confirmed that the binding and oligomerization of chemokines with GAGs are indispensable factors in the activity of chemokines *in vivo* (Proudfoot et al., 2003). Chemokines have been shown to be selective when interacting with GAGs. For example, for CCL5, the order of interaction strength is heparin, DS, HS, and CS, while mutant CCL5 has a reduced affinity for heparin. Studies have revealed that the main GAG-binding motifs on chemokines usually appear to be BBXB or BBBXXBX, where B and X represent a basic amino acid and any amino acid, respectively (Hileman et al., 1998). In addition, specific chemokine binding epitopes on GAGs have been found, such as the 2-O-sulfate group on the iduronic acid unit, which is necessary for formation of the GAG-dependent chemokine PF4 (Stringer and Gallagher, 1997).

### Receptor for Pathogens

The interaction of GAGs with specific proteins on the surface of a variety of pathogens, including viruses, bacteria, parasites and fungi, enables microorganisms to take the first step in establishing infection. Heparin-binding adhesins associated with intracellular pathogens, including gpB, gpC, and gpD of herpes simplex virus (HSV), gp120 of human immunodeficiency virus (HIV), herpesvirus filamentous hemagglutinin (FHA) of *Bordetella pertussis*, CS surface protein of *Plasmodium falciparum*, and the trypanosome adhesin penetrin, are likely the best studied proteins (Rostand and Esko, 1997). The protein sequences involved in the interaction between HSV and HS are conserved and functional in other alpha-herpesvirus glycoproteins. CD4 is the main receptor of the HIV-1 envelope glycoprotein gp120. The V3 and C4 domains of gp120 contain positively charged regions that can be aggregated in the oligomeric gp120 to form HS binding sites. Heparin and HS binding to Tat protein is also important in HIV-1 infection. Tat protein is one of the essential proteins for HIV-1 replication and is believed to play a role in triggering cell infection. The smallest heparin fragment involved in Tat binding is a hexasaccharide. Therefore, heparin is a “multi-target” compound that can affect different aspects of HIV infection (Capila and Linhardt, 2002). Dengue virus causes several human diseases, such as dengue fever, and infection is initiated by an interaction between the dengue E protein and protein, lipids, or carbohydrate host receptor(s). E protein, which is the major antigen, is involved in viral attachment and other biological processes. The structures and antibody binding sites of dengue virus E protein have been elucidated, and the results showed that specific carbohydrate residues with sulfation are common structures shared by CS-E and heparin and could be essential determinants for controlling dengue virus entry mediated by the E protein (Kato et al., 2010).

### Other Proteins

In addition to the above proteins, other proteins can also interact with GAGs, such as adhesion molecules, lipid or membrane-binding proteins, amyloid proteins and proteases. Cell adhesion molecules (CAMs) are a group of molecules that mediate contact and binding between cells or between cells and the extracellular matrix and can be divided into four main groups: the integrin family, the immunoglobulin superfamily, selectins (P, E, L) and cadherins. The interaction of GAGs with adhesion proteins involves a variety of physiological and pathological processes. For example, heparin tetrasaccharides specifically block the interactions of L- and P-selectins with antigen sialyl Lewis X-containing ligands, which show anti-inflammatory activity *in vivo* and prevent the adhesion of colon cancer cells to L- and P-selectin (Norgard-Sumnicht et al., 1993). Annexins belong to a homologous protein family that is closely related to the cell membrane, indicating that they are involved in various processes. Calcium-dependent lectin activity (Kojima et al., 1996) and/or binding to specific glycoproteins and binding of annexins IV, V, and VI to GAGs (including heparin, HS, or CS) have been reported. This interaction is not only based on the affinity of annexin to polyanions but also has structural specificity. The interaction between sucrose octathiosulfate and annexin V was found to be weaker than that of heparin-derived octasaccharide and annexin V combined with heparin and HS but not CS, which confirmed the specificity of the annexin V-heparin interaction (Ishitsuka et al., 1998). Apolipoprotein E (ApoE) is an important protein that can regulate lipid transport in human plasma and in the brain. The interaction between ApoE and cell-surface HSPGs is important for the liver to absorb lipoprotein residues. HSPGs on the cell surface can locate ApoE-enriched remnant lipoproteins to receptors through rapid correlation and separation (Futamura et al., 2005), facilitating lipoprotein uptake. The increased risk of AD associated with ApoE4 (Arg112, Arg158) appears to be associated with changes in amyloid-β (Aβ) homeostasis (O’Callaghan et al., 2014). The interaction between ApoE and low-density lipoprotein receptor (the LDLR family) and HSPG is also important for cell signaling events (Tai et al., 2016). The binding of heparin to neutrophil elastase, a serine protease, is involved in inflammation and pulmonary diseases, and targeting their binding site has led to discovering promising synthetic mimetics to treat cystic fibrosis (Morla et al., 2019).

Both specific and nonspecific interactions in protein/glycosaminoglycan associations reconcile the two opposing views that emphasize either the dominance of structural complementarity, similar to that encountered in protein/protein interactions, or electrostatic forces. An enormous structural heterogeneity makes the search for specific protein “recognition elements” an extremely challenging undertaking. At the same time, the polyanionic nature of GAGs highlights the role of charge density as an important determinant of affinity to a range of proteins. To date, a large number of GAG-binding proteins have been identified. New cases of GAGs interacting with proteins are being discovered, and the update of selected families of GAG binding proteins is summarized in Table 1. Due to the structural heterogeneity of GAGs, the negatively charged GAGs tend to attract proteins in a nonspecific manner, and due to the specificity of different protein binding sequences, it is reasonable to believe that there are still numerous unknown GAG-protein interactions waiting to be discovered.

**TABLE 1:** An update of selected families of GAG binding proteins is summarized.

Heparin-binding protein	Related diseases	Physiological/Pathological role	Characteristics of GAG binding	References
Spike glycoprotein	COVID-19	HS is a necessary co-factor for SARS-CoV-2 infection by interacting with both SARS-CoV-2 spike glycoprotein and ACE2 in the RBD.	HS transforms spinous process structure into open conformation through the interaction of receptor binding domain of spike glycoprotein of SARS-CoV-2, so as to promote the binding of ACE2	Clausen et al. (2020)
Tau, α-synuclein, and Aβ	Neurodegenerative diseases	Tau and α-synuclein aggregates bind HSPGs on the cell surface to mediate uptake and intracellular seeding	Tau aggregates require a precise gag structure with definite GAG fractions at the N- and 6-O- positions be substituted with sulfate groups, while the binding of α- synuclein to a Aβ is not so strict	Stopschinski et al. (2018)
HB-EGF	Cervical cancer	The expression of HB-EGF in tumor tissue was higher than that in stroma. Cervical cancer cells are the main source of HB-EGF.	HB-EGF is an important EGFR ligand in cervical cancer	Schrevel et al. (2017)
Transmembrane protein 184A (TMEM184A)	Angiogenesis	TMEM184A regulates angiogenesis by limiting endothelial cells proliferation and regulating extracellular growth	TMEM184A was identified as a heparin receptor in vascular cells. Heparin specifically binds to TMEM184A to induce anti-proliferative signaling	Farwell et al. (2017)
CXCL8	Inflammation	The binding of CXCL8 to GAGs on endothelial cell surfaces regulate neutrophil recruitment	Syndecan-4 (SDC4) was the potential proteoglycan co-receptor of CXCL8. CXCL8 binds to cell-surface HSPGs and leads to intracellular signal transduction in inflammatory tissue endothelium	Derler et al. (2017)
*Borrelia* glycosaminoglycan binding protein (Bgp)	Lyme disease caused by *Borrelia burgdorferi*	A variety of Bgp present in ***B***. *burgdorferi* provide functional redundancy during infection, which highlights the importance of GAGs as co-receptors for spirochetes adhering to host cells	The binding efficiency of Bgp to heparin was higher than that of chondroitin sulfate C	Schlachter et al. (2018)
FGF-2	Ischemic heart repair	FGF-2 promotes angiogenesis after MI. HSPG enhances cell adhesion, promotes the biological activity of FGF-2 in angiogenesis, and protects FGF-2 from enzymatic hydrolysis	The specific binding of HSPG to FGF-2 protein 6 times stronger than that of FGF-2 and heparin	Shi et al. (2019)
Receptor protein tyrosine phosphatase (RPTPσ)	Neural development and regeneration	RPTPσ has important functions in modulating neural development and regeneration	Both HS and CS bind to a series of lys residues located in the first Ig domain of RPTPσ. RPTPσ was aggregated by GAGs rich in 4,6-O-disulfated disaccharides	Katagiri et al. (2018)
C-type lectin 14a (CLEC14A)	Angiogenesis	CLEC14A is up-regulated during tumor angiogenesis and regulates endothelial cell migration and adhesion *in vitro* and angiogenesis *in vivo*	C-type lectin domain of CLEC14A binds 1:1 to heparin with nanomolar affinity. CLEC14A prefers highly charged polysaccharides	Sandoval et al. (2020)
Keratinocyte-derived chemokine (KC or mCXCL1) and macrophage inflammatory protein 2 (MIP2 or mCXCL2)	Inflammation	KC and MIP2 play important roles in transporting neutrophils to infected and injured sites	Different combinations of residues from the N-loop, 40s turn, β_3_-strand, and C-terminal helix form a binding surface within a monomer and both conserved residues. The binding interaction is mediated by both conserved residues and residues specific to chemokines	Sepuru et al. (2018)
Pre-S region of HBV envelope proteins	Hepatitis B	The human hepatic cell-binding site (i.e., the sodium taurocholate co-transporting polypeptide (NTCP)-binding site, with myristoylated pre-S1 (2–47)) and the low pH-dependent fusogenic domain (pre-S1 (9–24)) are required for targeting and endosomal escape, respectively	A novel heparin-binding site (pre-S1 (30–42)) in the N-terminal half of the pre-S1 region may interact with cell-surface HSPG. The amino acid residues Asp-31, Trp-32, and Asp-33 are essential for heparin-binding activity	Liu et al. (2018)

In summary, GAGs interplay with a wide range of important proteins. These proteins belong to different families and play various roles in physiological or pathological processes. Selectivity is the key when studying the binding between GAGs and proteins. Because the ionic force between negative charges of GAGs and positive charges on proteins is the basis of their interaction, abnormally highly charged GAGs, such as oversulfated CS or oversulfated HS, usually bind to basic proteins with high affinity but little specificity, which will cause uncontrollable side effects if being used as drugs. Elucidating and designing defined GAG sequence that specifically interacts to certain protein will be the only plausible way to develop promising new GAG therapeutics.

## Analytical Tools and Approaches for Characterization of GAG-Protein Interactions

Obviously, safe and effective therapeutic intervention for diseases associated with GAGs depends on the selection of appropriate structures with the desired characteristics and a lack of harmful effects. For example, when using heparin or related compounds to treat COVID-19, the candidate drugs must have the ability to hinder the ACE2/S-Protein interaction with few deleterious effects (e.g., the HIT caused by binding to PF4). This work can be greatly facilitated by analytical tools that provide detailed information on the interactions between candidate drugs and their therapeutic targets.

Currently, numerous analytical approaches have been developed and applied to reveal the molecular mechanism and binding sequence of GAG-protein complexes (Yang and Chi, 2017). Affinity approaches, such as affinity chromatography, surface plasmon resonance (SPR) and isothermal titration calorimetry (ITC), are used to measure the binding strength between GAGs and proteins. NMR spectroscopy and X-ray crystallography can present extremely valuable information about GAG-protein interactions, providing structural and conformational data that are useful in identifying the precise contact points between interacting molecules. The microarray platform and molecular docking are powerful tools to screen protein interactions against large GAG structure libraries, and intricate dynamic details of molecular-level events can be visualized with a relatively small time and cost investment. However, the structural heterogeneity of GAGs and the extensive glycosylation of the proteins involved still make discovery of the specificity of the binding sequence challenging.

MS techniques have several unique advantages in the characterization of GAG-protein complexes due to their superior sensitivity, tolerance of lower sample purity and ability to characterize amino acid/sugar residues and modification. Ion mobility spectroscopy (IMS) (Gray et al., 2016), hydroxyl radical footprinting (HRF) (Li et al., 2015) and cross-linking MS (Yang et al., 2012) have been used to study the interactions between GAGs and proteins. Recently, native mass spectrometry has been used as a tool to support mechanistic study of drug/therapeutic target interactions (Tong and Wang, 2018). Using gas-phase ion manipulation (limited charge reduction) and molecular modeling to supplement native MS has allowed obtainment of meaningful information about the complex formed by ACE2 and S protein and the role of heparin in destroying ACE2/RBD binding (Yang et al., 2020). A top-down approach was used to maintain the chemical diversity of heparin by allowing complex long chains to interact with the target protein. After enzymolysis, the protein-binding heparin chains were analyzed using size exclusion chromatography with online mass spectrometry detection (SEC/MS) (Niu et al., 2020), which revealed the oligomers that were not cleaved by lysis due to their binding to the protein and enabled characterization of chain length and sulfate and acetyl groups. Some of the latest mass spectrometry techniques and their applications in GAG-protein interactions are shown in Table 2.

**TABLE 2:** The recent advances in mass spectrometry based analytical tools is summarized.

Method	Principle	Applications	References
Mass spectrometry combined with gas-phase ion manipulation technique	Intact macromolecules or macromolecular complexes are directly ionized from non-denaturing solvent, and key noncovalent interactions that hold the complexes together can be preserved for MS analysis in the gas phase	Characterizing biomacromolecular structure and interactions under physiologicalconditions. For example, obtaining meaningful information about the complex formed by ACE2 and S protein, and the role of heparin in disrupting ACE2/RBD binding	Yang et al. (2020)
SEC-MS	When the SEC-MS system is applied to heparin, a series of oligomers with different sulfation levels can be generated	Enzymatic lysis was used to product the proteinbound chains, then mass spectrometry detection (SEC/MS) can detect the tight association with the protein, including the characterization, oligomer length and the number of incorporated sulfate and acetyl groups	Niu et al. (2020)
Cross-linking MS	Cross-linking with MS approach has been recently recognized as a powerful tool to study protein-protein interaction. It can also study GAG-protein interactions by “locking” binding motifs together through covalently cross-linking carboxyl groups of GAGs to amine side chains of protein	The cross-linking technique locks down the binding motifs of GAGs and proteins through covalent reactions. For example, the carboxyl groups of GAGs can be activated by EDC and sulfo-NHS, then form a zero-length linkage with the amine side chains of proteins. After digestion by protease, LC/MS/MS analysis showed that the binding motif was oligosaccharide peptide conjugate	Yugandhar et al. (2020)
Limited Proteolysis in the absence of denaturation, heparin-Affinity chromatography, and high-resolution LC-MS/MS proteomics (LPHAMS)	By using suboptimal conditions for proteolysis, limited cleavage occurs at exposed hinges or loops, resulting in the release of intact protein domains. Liberated domains by chromatography on heparin-affinity resin would identify potential HSBPs and enrich HS-binding domains	Identification and characterization of membrane-anchored and extracellular proteins that bind HS. Application of LPHAMS has led to the identification of large number of HSBPs. In many cases, this method reveals subdomains that promote HS binding	Sandoval et al. (2020)

Another emerging field is developing computational tools to facilitate the study of GAG-protein interactions. Unlike proteins, GAGs are highly charged and highly flexible at the aspect of confirmation. Therefore, it is difficult to obtain high quality cocrystals of GAG-protein complexes. Computational approaches provided an alternative way to predict the binding patterns and residues contributed the binding. A systematic study has been carried out by computationally characterizing all known GAG-protein bindings from the Protein Data Bank, which proved the feasibility of the computational methodology (Bojarski et al., 2019). Furthermore, a GAG-Dock methodology has been developed to evaluate the binding between various GAG ligands and receptors that are essential in axonal growth, and their plausible structures were provided (Griffith et al., 2017). Our group has also applied the molecular docking approach to explain the pharmacokinetic behavior of heparin in diabetic patients by simulating the binding of heparin and glycated human serum albumin (Qiu et al., 2020). Computational study of GAG-protein binding is also useful in developing potential therapeutics, such as sulfated small molecules mimicking the function of GAGs (Nagarajan et al., 2017). Indeed, the computational methodology has become extremely useful and easily accessible to non-computational researchers (Sankaranarayanan et al., 2018).

## Therapeutics Targeting Specific GAG-Protein Interactions

**FIGURE 3:**
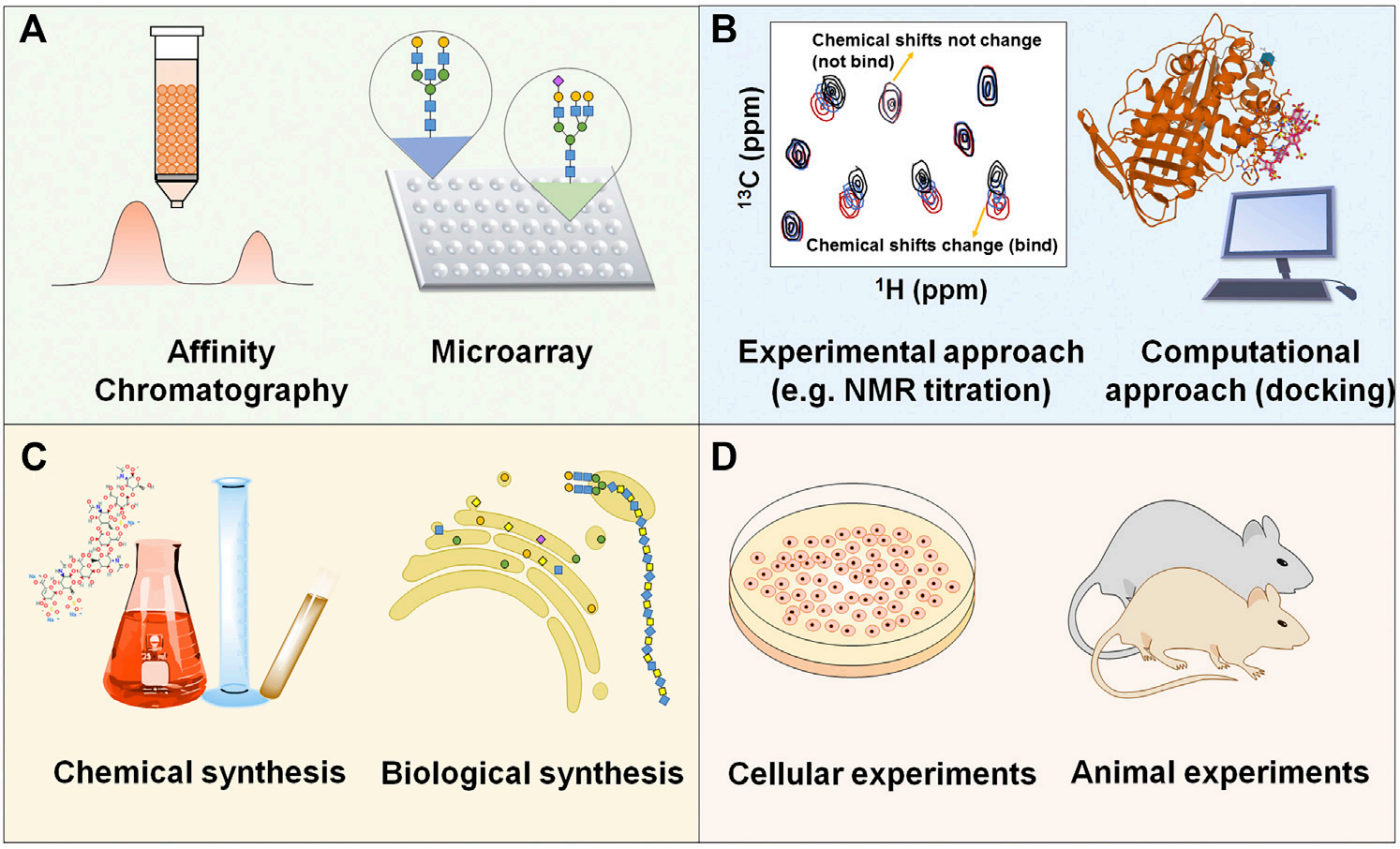
Schematic strategy of developing novel therapeutics based on the specific interaction between GAGs and proteins **(A)** Discovering GAG-protein binding. **(B)** Elucidating the molecular mechanism between the binding. **(C)** Synthesizing specific GAG oligosaccharides or analogs. **(D)** Evaluating the efficacy and toxicity *in vitro* and *in vivo*.

The eventual goal of studying interaction between GAGs and proteins is to develop novel therapeutics from this promising but inadequately explored field. The schematic strategy is shown in Figure 3. Actually, some therapeutics targeting specific GAG-protein interactions, including GAG oligosaccharides and synthetic analogs, removal or modification of GAGs by enzymes, exogenous heparin/HS or synthetic GAG mimetics as competitive inhibitors, cationic proteins and polymers as HS antagonists, and small molecule antagonists of heparin and HS, are currently being developed or have been applied to treat related diseases.

The application of heparin oligosaccharides and synthetic analogs is an important aspect of the clinical treatment of many diseases. HP and LMWH have long been used as anticoagulants (Hirsh et al., 2001). The synthetic pentasaccharide Arixtra (Fondaparinux) binds to AT and has better efficacy at low doses (Walenga et al., 2002). Additionally, some therapeutic applications of heparin and its derivatives beyond anticoagulation have been explored in patients with bronchial asthma, chronic obstructive pulmonary disease (COPD) and cancer. The anti-inflammatory, antioxidant, and lysogenic effects of heparin administered via the inhalation pathway may alter the progression of COPD and asthma (Shute et al., 2018).

Several different strategies to target the interactions of HS and proteins have been explored, including HS removal or modification by enzymes (Rek et al., 2009). Some heparin enzymes (such as bacterial heparinases and mammalian endosulfatases) have been shown to be potential inhibitors of HS-protein interactions. Heparinase therapy has also been used to inhibit tumor growth/metastasis and amyloid-related diseases. Cells treated with heparinase can resist the attachment or entry of several HS-binding pathogens, including viruses, bacteria, and parasites (Weiss et al., 2017).

Another way to inhibit HS-protein interactions is to use exogenous heparin/HS or synthetic GAG analogs as competitive inhibitors. Exogenous addition of heparin and HS chains can inhibit infection of host cells with HS-binding pathogens, such as HSV, HPV, hepatitis B and various bacteria. Additionally, cancer cell growth and metastasis can be blocked by HS and heparin. LMWHs and HS mimetics (Lee et al., 1999), such as rhamnan sulfate, have shown anticancer and antiviral activity, which was promising when tested *in vitro*.

Cationic proteins and foldamers have been used as antagonists of HS-protein interactions. These molecules depend on electrostatic interactions between their positively charged functional groups and the high anion sulfate and carboxylic acid groups of heparin and HS. Lactoferrin (Lonnerdal and Iyer, 1995) has been tested to neutralize heparin and antagonize certain HS–protein interactions. Protamine (Taylor and Folkman, 1982) has been demonstrated to be a potent antagonist of the GAG-protein interaction and has been used clinically to reverse anticoagulants.

Certain small molecule drugs have been developed as HS-protein antagonists due to their specific characteristics and advantages. For example, a dispirotripiperazine derivative (DSTP 27) (Schmidtke et al., 2003) was found to bind cell surface HS and inhibit attachment and absorption of some viruses and to block HS-dependent viral attachment of an HPV virus in the long term.

## Conclusion and Marks

GAGs are involved in a large number of biological processes and play an important role in growth and development, maintaining homeostasis and resisting disease. GAGs affect cell adhesion, migration, signal transduction and other biological activities through interactions with proteins, such as growth factors and adhesion factors, thereby affecting numerous physiological activities. Due to the diversity of the types and functions of the proteins that interact with GAGs, GAGs exert a variety of biological functions. The occurrence and development of many diseases, from the invasion of pathogens to the occurrence and development of tumors, are related to GAGs. Elucidation of the specific sequence and mechanism by which GAGs interact with proteins is essential for finding novel therapeutics targeting specific GAG-protein interactions.

## Insights and Future

The research, development and market of carbohydrate-based drugs, especially GAG-based drugs, are far behind the protein-based drugs. Except for heparin drugs as anticoagulants, few GAGs have been widely used in clinic, although GAGs exhibit a wide range of bioactivities. However, the situation is changing now. With the advances of analytical tools and synthetic/biosynthetic approaches, identifying specific sequence and obtaining sufficient structure uniform GAG oligosaccharides become feasible. In the next five to ten years, we can expect quite a few GAG or GAG mimetics proceed to clinical trials. It will boost the GAG study and lead to new solutions for diseases that are difficult to be cured by current small molecule or protein drugs.
